# An fNIRS Study of Applicability of the Unity–Diversity Model of Executive Functions in Preschoolers

**DOI:** 10.3390/brainsci12121722

**Published:** 2022-12-16

**Authors:** Sha Xie, Chaohui Gong, Jiahao Lu, Hao Zhang, Dandan Wu, Xinli Chi, Hui Li, Chunqi Chang

**Affiliations:** 1 Faculty of Education, Shenzhen University, Shenzhen 518060, China; 2 School of Biomedical Engineering, Health Science Center, Shenzhen University, Shenzhen 518060, China; 3 Department of Early Childhood Education, The Education University of Hong Kong, Hong Kong SAR, China; 4 School of Psychology, Shenzhen University, Shenzhen 518060, China; 5 Shanghai Institute of Early Childhood Education, Shanghai Normal University, Shanghai 200233, China; 6 Macquarie School of Education, Macquarie University, Sydney 2109, Australia; 7 Peng Cheng Laboratory, Nanshan District, Shenzhen 518055, China

**Keywords:** executive function, early childhood, fNIRS, working memory, cognitive shifting, inhibitory control

## Abstract

Executive function (EF) includes a set of higher-order abilities that control one’s actions and thoughts consciously and has a protracted developmental trajectory that parallels the maturation of the frontal lobes, which develop speedily over the preschool period. To fully understand the development of EF in preschoolers, this study examined the relationship among the three domains of executive function (cognitive shifting, inhibitory control, and working memory) to test the applicability of the unity–diversity model in preschoolers using both behavioral and fNIRS approaches. Altogether, 58 Chinese preschoolers (34 boys, 24 girls, *M_age_* = 5.86 years, *SD* = 0.53, age range = 4.83–6.67 years) were administered the *Dimensional Card Change Sort* (DCCS), go/no-go, and missing scan task. Their brain activations in the prefrontal cortex during the tasks were examined using fNIRS. First, the behavioral results indicated that the missing scan task scores (working memory) correlated with the DCCS (cognitive shifting) and go/no-go tasks (inhibitory control). However, the latter two did not correlate with each other. Second, the fNIRS results demonstrated that the prefrontal activations during the working memory task correlated with those in the same regions during the cognitive shifting and inhibitory control tasks. However, the latter two still did not correlate. The behavioral and neuroimaging evidence jointly indicates that the unity–diversity model of EF does apply to Chinese preschoolers.

## 1. Introduction

Executive function (EF) includes a set of higher-order abilities to control one’s actions and thoughts consciously [1,2] and is related to the prefrontal cortex, a region important for top-down control [3]. Therefore, EF has a protracted developmental trajectory that parallels the maturation of the frontal lobes, which develop speedily over the preschool period and continue to mature throughout adolescence and adulthood [4,5]. Many models of EF have emerged over the past decades, and the most prominent one was proposed by Akira Miyake and his colleagues [6,7], which emphasizes the unity and diversity of those executive processes. According to this model, there are some common executive processes (the unity of EF) and some unique to the specific EF components, including cognitive shifting, inhibitory control, and working memory (the diversity of EF). However, a recent meta-analysis of existing studies could only confirm that in school-aged children (6 years or up), there were partially separable but partially overlapping executive processes at a neural level [8]. This conclusion indicated that the unity and diversity model of EF could only apply to school-aged children, leaving preschoolers (ages 4–6) unexplored. To fill this gap, this study explored the unity and diversity of EF in the preschool years using both behavioral and neuroimaging evidence.

### 1.1. Behavioral Study of the Three Components of EF

It is widely believed that EF consists of three components: (1) *shifting* or cognitive shifting: the ability to switch flexibly between tasks or mental states; (2) *inhibition* or inhibitory control: the ability to deliberately override dominant or prepotent responses; and (3) *updating* or working memory: the ability to monitor and add/delete working memory contents [7]. Using confirmatory factor analysis, the existing studies have suggested some continuity in the three-factor EF from school children to adults [9,10,11]. However, studies on preschoolers have suggested a unitary [10,11,12,13] or a two-factor structure with inhibition and working memory as separate but correlated factors [14,15,16]. Moreover, the exact factor structure seems to vary over age and task (quantity and content), demonstrating mixed results [17]. and a systematic review of the unity and diversity of executive functions supported the increasing multidimensionality of executive functions over the course of development. Still, the findings suggested that it might derive from methodological differences between child and adult studies, such as the number of indicators used per construct in measurement models [18].

Despite the mixed findings on preschoolers, it is generally suggested that key EF components emerge during the first three years of life, which include some simple skills (i.e., holding information in mind), and are integrated into the complex processes (inhibitory control, working memory, and cognitive shifting). This development is hierarchical and characterizes the maturation of EF abilities [19]. Thus, given the rapid growth of EF during early childhood (ages 4–6), it is critical to confirm whether the unity and diversity of EF apply to preschoolers to clarify conceptual and methodological ambiguity, guide future research, and inform early intervention [20].

As for the experimental designs of the three components, previous research used different assessment instruments to measure young children’s cognitive shifting, inhibitory control, and working memory. First, the Dimensional Change Card Sort (DCCS) is a widely used and age-appropriate tool to measure the development of cognitive shifting in young children [21]. Researchers generally found that 3 year olds tend to stick to rules in the pre-switch rules but show difficulty with the post-switch rules, while 4- and 5-year-old children correctly sort the cards according to the post-switch rules [5,22]. However, more recent studies found that even some 4 and 5 year olds might fail to switch rules [23], making it interesting to explore the developmental patterns of cognitive shifting using the DCCS paradigm. Second, the go/no-go and Stroop-like tasks have been used to index young children’s inhibitory control with various adaptations [24,25,26]. However, the Stroop-like tasks required young children’s understanding of the spoken language or the written word to perform the task; thus, the go/no-go task was more suitable for young children [5]. Third, the experiment paradigm for young children’s working memory had more variations, given the complexity of the concept of working memory itself. Previous studies have used the Memory for Picture of Wechsler Intelligence Scale for Children [24], the change detection task [27], and the missing scan task [28] to examine young children’s working memory. Given the focus on working memory capacity, the missing scan task, adapted from the Buschke missing scan methodology, was used in the current study. Finally, the previous studies found that children’s performance in cognitive shifting was correlated with that in inhibitory control [6] and working memory [29], while performance in inhibitory control was correlated with that in working memory [24]. However, very few studies have systematically examined the relationship among the three executive processes, a research gap the current study intended to explore. Thus, Hypothesis 1 is proposed: There would be significant correlations between the behavioral performances in the three EF tasks.

### 1.2. The Neural Correlates of Executive Function

Advances in behavioral and neuroimaging approaches have provided evidence that EF is located not only in the prefrontal cortex (PFC) but also in areas of the frontoparietal network, such as the dorsolateral prefrontal cortex (DLPFC), ventrolateral prefrontal cortex (VLPFC), and posterior parietal cortex (PPC), as well as subcortical regions [30,31,32,33]. Regarding the neural basis of the three EF factors, the existing studies also identified the regions activated during the respective tasks. First, for preschoolers’ cognitive shifting, longitudinal fNIRS studies on the prefrontal cortex activation revealed that prefrontal cortex activation plays a vital role in successful switching during the Dimensional Change Card Sort (DCCS) task [34]. Furthermore, using the fNIRS technique, a mindfulness training study revealed that behavioral changes during the DCCS task are related to changes in the DLPFC [35]. Nevertheless, fMRI studies during the DCCS tasks showed that the functional network of cognitive shifting is still developing after the preschool period: the LPFC might be significantly more connected with the inferior parietal cortex and subcortical regions in adults than in children [36]. Second, for children’s inhibitory control, an fNIRS study showed an age-independent effect in the right PFC and an age-dependent effect in the left orbitofrontal cortex (IOFC) [37]. Furthermore, a comparative study using fNIRS imaging revealed that children and adults might have different patterns: children had stronger parietal coherence in short-range functional connectivity in the right frontal and right parietal cortices, but adults showed long-range functional connectivity between bilateral frontal and parietal areas [26]. Third, for children’s working memory, neuroimaging studies have shown that activation in the lateral prefrontal cortex, right premotor areas, caudal superior frontal sulcus, and right inferior prefrontal gyrus were detected during visuospatial working memory tasks [38,39,40]. In addition, the existing fNIRS studies revealed that preschoolers’ prefrontal and parietal regions were activated during working memory tasks [27,41]. The previous studies, however, examined the changes in oxygenated hemoglobin (HbO) in the prefrontal regions for the three executive processes separately, leaving their interrelated relationships underexplored. This study is dedicated to filling this gap.

### 1.3. The Unity–Diversity Framework of Executive Function

Miyake and Friedman (2012) reviewed the existing studies to comprehend the nature of individual differences in EF and its cognitive and biological foundations. Based on the review, they developed a new theoretical framework: the unity–diversity framework. This framework proposes that individual differences in EF show both unity (as there are some common executive processes) and diversity (as there are some processes unique to the specific EF components, such as cognitive shifting, inhibitory control, and working memory). Furthermore, they believe this framework reflects substantial genetic contributions and demonstrates developmental stability.

The existing neuroimaging studies have supported this unity–diversity model in school-aged children and adults. For example, meta-analyses of fMRI data found both separable executive processes (i.e., diversity) [42] and a common activation indicative of an overarching EF network (i.e., unity) [43,44]. The existence of a superordinate cognitive control network involving dorsolateral prefrontal, anterior cingulate, and parietal cortices that supports a broad range of executive functions was confirmed in healthy individuals aged 18–60 using quantitative meta-analytic methods [44]. Neuroimaging studies in school-aged children have generally focused on the emergence and maturation of specific EF processes examined separately but suggested different developmental trajectories as indicated by age-related activation changes [45,46,47,48]. A recent meta-analysis confirmed that school-aged children (ages 6–12) had partially separable but partially overlapping executive processes at a neural level, indicating that the unity and diversity model of EF applies to children of this age [8], with significant bilateral activation in frontoparietal areas and regions of the supplementary motor area across, suggesting common executive components. However, no neuroimaging studies have ever explored the applicability of this model to preschoolers (ages 4–6), leaving a research gap to be addressed by this study. Given the neural evidence for both the unity and diversity models, Hypotheses 2 and 3 are proposed. There would be significant correlations between the prefrontal activations in the three EF tasks (Hypothesis 2). There would be significant differences in the prefrontal activations between the three EF tasks (Hypothesis 3).

### 1.4. The Present Study

Preschoolers may activate the lateral prefrontal regions during EF tasks that tap their cognitive shifting, inhibitory control, and working memory. According to the unity–diversity model [7], the three executive processes might be highly correlated at the neural level, even during preschool years. As there has been no neuroimaging evidence to support the applicability of this unity–diversity model in preschoolers, this study is dedicated to examining the unity and diversity of EF in young children using both behavioral and neuroimaging approaches. The advantage of the fNIRS in providing a more spatially resolved image of brain activity compared to EEG, and being portable, and having a high sampling rate compared to fMRI [49], make it suitable for studying the brain activities of young children. Furthermore, the behavioral experiments have been validated in previous studies [26,27,50,51] using the fNIRS in preschoolers, making them plausible to examine the characteristics of the three executive processes. In particular, the Dimensional Change Card Sort (DCCS) [21] task is used to measure cognitive shifting, the go/no-go task [52] to measure inhibitory control, and the missing scan task [28] to measure working memory. Meanwhile, the concentration changes of oxygenated hemoglobin (HbO) and deoxygenated hemoglobin (HbR) in the dorsolateral and ventrolateral prefrontal activations are assessed using fNIRS. 

## 2. Materials and Methods

### 2.1. Participants

Altogether, 62 right-handed Chinese preschoolers participated in this study. These participants had no known developmental disorders. Four participants were excluded from formal analysis due to failure to finish the tasks, resulting in a final sample of 58 children. Among these children, 34 were boys, and 24 were girls, *M_age_* = 5.86 years, *SD* = 0.53, age range = 4.83–6.67 years.

### 2.2. Behavioral Task

The participants were invited to perform the three tasks to measure their cognitive shifting, inhibitory control, and working memory: DCCS, go/no-go, and missing scan tasks. All the tasks were computerized using Psychophysics Toolbox extensions and displayed on a 55.35 cm × 31.13 cm Dell monitor.

#### 2.2.1. Dimensional Change Card Sort Task

The DCCS task was originally developed by Zelazo et al. [21] and modified by Xie et al. [35] to accommodate the block design of fNIRS, which was suitable for children aged 3 to 6 years old [23]. It was employed to measure cognitive shifting in this study. Two target cards with two dimensions (i.e., a red rabbit and a blue boat) were used as stimuli and displayed in the upper center of the screen. One test card (i.e., a blue rabbit or a red boat) appeared on the lower center of the screen, which matched the test cards on one dimension but not in the other (color or shape). The participants performed three consecutive test blocks, and each block consisted of a pre-switch (25 s) and post-switch phase (25 s). A line of instruction in grey appeared on the bottom of the screen to remind the experimenter of the beginning or end of the task. In the pre-switch phase, they were asked to sort the cards according to one rule (e.g., color), and in the post-switch phase, they were instructed to sort the cards according to the second rule (e.g., shape). The rule order for the three blocks was fixed and applied to all the participants to control for learning effects: (1) color → shape; (2) shape → color; and (3) color → shape. The participants pointed to the target card, and the experimenter pressed the key to record the answers. 

**Figure 1:**
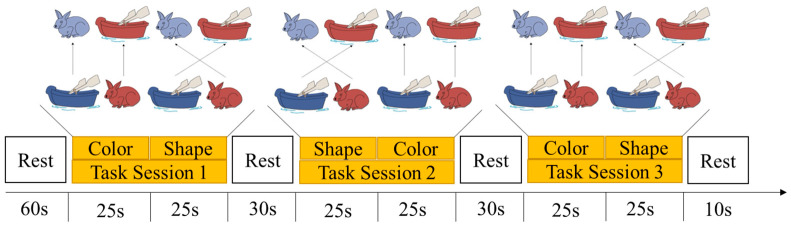
Task paradigm of DCCS Task.

The aggregate number of correct responses in all the blocks was calculated as a measure to index total performance. However, the experimenter recorded participants’ responses, and response time could not accurately reflect children’s performance. Thus, the accuracy rate was calculated by dividing the correct trials by the total trials and was used for subsequent analysis. The task paradigm of DCCS is shown in Figure 1. 

#### 2.2.2. Go/No-Go Task

**Figure 2:**
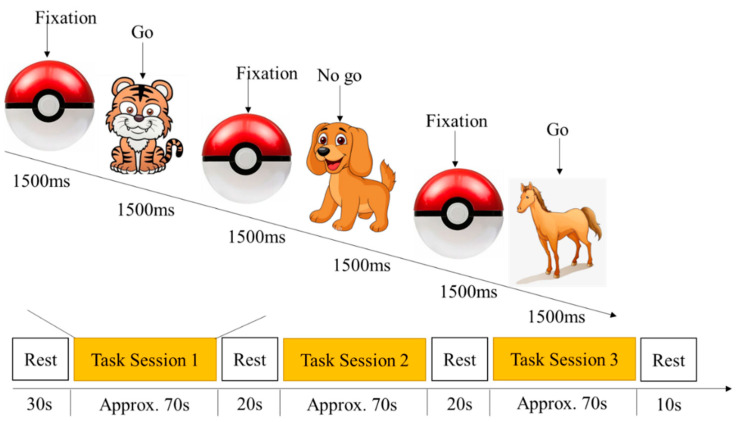
Task paradigm of Go/No-Go Task.

The go/no-go task was modified from Lahat et al.’s [52] paradigm to measure children’s inhibitory control, which has good validity and well-mapped neural bases [53]. In each trial, an animal stimulus (cow, horse, tiger, or dog) was presented at the center of the screen. The participants were instructed to press the “space” key on the keyboard as soon as they saw each animal (go stimuli) except for the dog (no-go stimulus). They were told not to press when they saw the dog. In the practice session, there were four go trials and four no-go trials in the training session, where children were reminded of the rules should they respond incorrectly. The task consisted of 30 go trials and 30 no-go trials divided into three task blocks, with 10 go trials and 10 no-go trials randomly distributed within each block. The participants performed three consecutive test blocks with rest phases in between. The accuracy rate by dividing the correct trials by the total trials was used for subsequent analysis. The task paradigm of go/no-go is shown in Figure 2.

#### 2.2.3. Missing Scan Task

**Figure 3:**
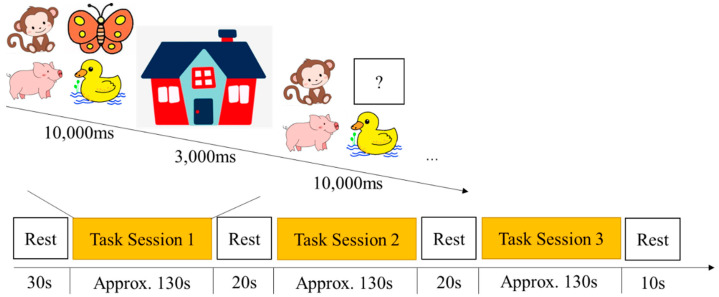
Task paradigm of Missing Scan Task.

The missing scan task was modified from Roman’s task, which is suitable for measuring working memory capacity for preschoolers (ages 3–6) [28]. It was adapted into a block to fit the fNIRS experiment paradigm in this study. A total of 30 animal figures were used as test stimuli, such as monkey, butterfly, duck, and pig. In each trial, four animals appeared on the screen for 10 s, and the participants were instructed to name pictures of each animal to prevent the need to learn new vocabulary. Then, the four animals disappeared into a “house” for 3 s. After that, three animals reappeared on the screen, and the participants were instructed to verbally respond to the name of the missing animal in 10 s before the next set of animals appeared on the screen. The experimenter recorded the participants’ responses using the keyboard. There were two trials in the practice session to ensure the participants understood the test rules, and there were five trials in each one of the test blocks. The participants performed three consecutive test blocks with rest phases in between. The task paradigm of the missing scan is shown in Figure 3 (Figure 3). The accuracy rate by dividing the correct trials by the total trials was used for subsequent analysis.

### 2.3. Functional Near-Infrared Spectroscopy Recordings

**Figure 4:**
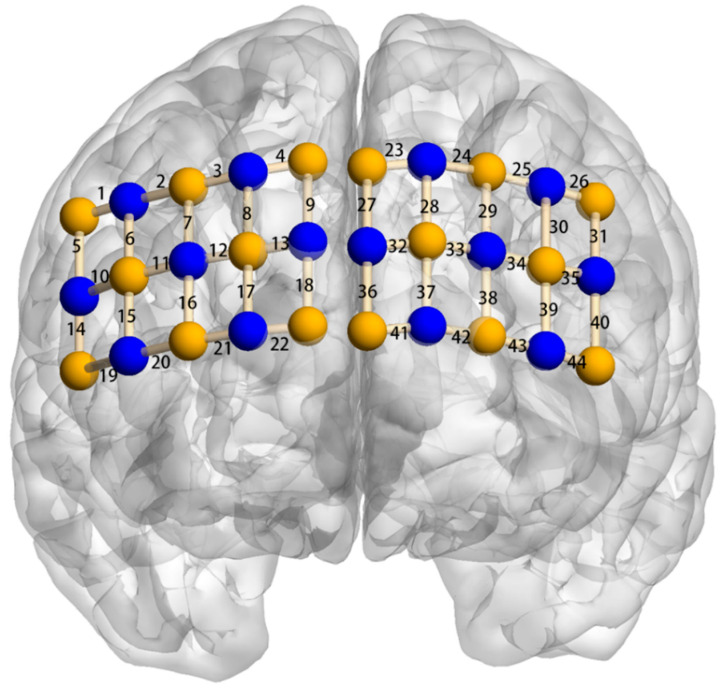
Localization of regions of interest. *Note.* Right ventrolateral prefrontal cortex (VLPFC): channel 16, 17, 21, 22; left VLPFC: channel 38, 39, 42, 43; right dorsolateral prefrontal cortex (DLPFC): channel 3, 4, 7, 8, 9, 12, 13; left DLPFC: channel 24, 25, 28, 29, 30, 33, 34; right posterior superior frontal cortex (PSFC): channel 1, 2, 5, 6; left PSFC: channel 26, 31; right temporal cortex (TC): channel 10, 11, 14, 15, 19, 20; left TC: channel 35, 40, 44; and medial prefrontal cortex (MPFC): channel 18, 23, 27, 32, 36, 37, 41.

A multichannel fNIRS system (OxyMon MK III, Artinis, The Netherlands) was used to measure the concentration changes of oxygenated hemoglobin (HbO) and deoxygenated hemoglobin (HbR) at wavelengths of 762 and 846 nm in the participants. Following the study design of previous studies on young children’s EF [54,55], the fNIRS probe consisted of 30 optodes using a 3 × 10 light level stencil located in the forehead, which constituted 44 channels to cover the frontal area. Each channel consisted of one emitter and one detector optode, with a 2.5 cm distance. To ensure consistent light level array positions for all participants, the lower middle of the array was positioned at the Fpz position, which is consistent with the 10–20 measurement system. Accordingly, the region of interest (ROI) was the left ventrolateral prefrontal cortex (VLPFC), right VLPFC, left dorsolateral prefrontal cortex (DLPFC), right DLPFC, left posterior superior frontal cortex (PSFC), right PSFC, left temporal cortex (TC), right TC, and medial prefrontal cortex (MPFC) (see Figure 4). Previous studies have shown that the frontal area is actively involved in EF [35,51]. The sampling rate was set at 50 Hz for data acquisition. A differential pathlength factor (DPF) value was calculated for each participant according to the formula (DPF = 4.99 + 0.0678 × Age^0.814^) based on their age [56].

### 2.4. Procedure

The study was conducted in accordance with the principles of the Declaration of Helsinki and approved by the University Ethics Committee of the first author. All the parents of the participating children provided written consent and were informed verbally of the research purpose and the safety of the fNIRS experiment. Then, each child was invited into a quiet room in the preschool to receive the EF tasks. An experienced NIRS technician placed the NIRS cap on the child while an experienced preschool teacher engaged in book reading with the child. Before data collection, the OxySoft (data collection software for the equipment OxyMon) showed the signal quality that indicated whether the optical coupling between the optodes and the scalp was good. If the coupling was not good, the experimenter used a tool to move the participant’s hair to ensure that the optode was pressed against the scalp. During data collection, OxSoft showed real-time signal quality. In the case of channels with low signal quality, the experimenter marked down that channel to be removed from later data analysis.

### 2.5. Analytic Plan

First, participants’ behavioral results were exported and analyzed in Matlab. Then, descriptive and correlational analyses were conducted to examine the relationship across the three tasks to examine Hypothesis 1.

Next, the fNIRS data were preprocessed. Due to higher sensitivity to changes in cerebral blood flow [57,58], higher signal-to-noise ratio [57], and retest reliability [59], we focused on the blood oxygen concentration (HbO). The HbO data were first visually inspected to assess the quality of the data. The channels with high-frequency signal interference caused by bad optical coupling between the optode and the scalp, as well as head movements, were removed before formal analysis [60]. Next, the NIRS-KIT software [61] was used to perform first-order baseline correction on the HbO. Accordingly, the DTTR algorithm was used to remove motion artifacts [62]. Slow drifts and high-frequency noises were reduced using the bandpass filter (third-order Butterworth filter) with cut-off frequencies of 0.01–0.09 Hz [63]. After that, the difference in the average changes in HbO during the corresponding rest and task phases in each task was used as the dependent variable in the following analyses. To increase the signal-to-noise ratio, the 44 channels were averaged into nine ROIs, where the time-series data were averaged within each ROI. Finally, the correlations of activations between the differences in average changes in HbO across tasks were assessed.

Finally, whether the activations in the ROIs differed across tasks was examined to examine Hypotheses 2 and 3. The difference in the average changes in HbO during the corresponding rest and task phases in each task was used as the dependent variable in this analysis. A 3 × 9 ANOVA was conducted with the three tasks and nine regions for HbO.

## 3. Results

### 3.1. Behavioral Results

The results of descriptive analysis and correlation analysis are shown in Table 1. On average, the participants had a 97% (*SD* = 0.00), 93% (*SD* = 0.01), and 62% (*SD* = 0.05) correct rate in the cognitive shifting, inhibitory control, and working memory tasks, respectively. Furthermore, the participant’s performance in the missing scan task was correlated with their performance in DCCS (*r* = 0.26, *p* < 0.05) and go/no-go task (*r* = 0.53, *p* < 0.001). However, the DCCS was not significantly correlated with the go/no-go task (*r* = 0.13, *p* > 0.05). Then, partial correlational analyses were conducted after controlling for children’s age, and the performance in the missing scan correlated with the go/no-go task (*r* = 0.42, *p* < 0.01). Therefore, Hypothesis 1 is not supported by this study.

### 3.2. The fNIRS Results

Results for the changes in HbO are depicted in Table 2. Next, correlations of the prefrontal activations in the nine ROIs across the three tasks were examined. The correlations between the DCCS and go/no-go tasks are depicted in Table 1. Results showed that for the tasks DCCS and go/no-go, there were no significant correlations in the same region (*p*s > 0.05), but there were significant correlations in the left VLPFC during DCCS and left DLPFC during the go/no-go task (*r*(58) = −0.30, *p* < 0.05), the left VLPFC during DCCS and left TC during the go/no-go task (*r*(58) = −0.28, *p* < 0.05), the left PSFC during DCCS and MFPC during the go/no-go task (*r*(58) = 0.29, *p* < 0.05), and the leftTC during DCCS and MFPC during the go/no-go task (*r*(58) = 0.30, *p* < 0.05). Results showed that for the tasks DCCS and missing scan, there were significant correlations in the same region of left VLPFC (*r*(58) = 0.28, *p* < 0.05) and right DLPFC (*r*(58) = 0.38, *p* < 0.001), as well as significant correlations in the left VLPFC during DCCS and right VLPFC during the missing scan task (*r*(58) = 0.37, *p* < 0.05), the left VLPFC during DCCS and right DLPFC during the missing scan task (*r*(58) = 0.32, *p* < 0.05), the right PSFC during DCCS and right DLPFC during the missing scan task (*r*(58) = −0.29, *p* < 0.05), and the right TC during DCCS and left DLPFC during the missing scan task (*r*(58) = 0.29, *p* < 0.05). The results showed that there were significant correlations in the same region of left DLPFC (*r*(58) = 0.28, *p* < 0.05), as well as significant correlations in the left PSFC during the go/no-go and right TC during the missing scan task (*r*(58) = −0.48, *p* < 0.01), the right PSFC during the go/no-go and left PSFC during the missing scan task (*r*(58) = 038, *p* < 0.05), the right PSFC during the go/no-go and left TC during the missing scan task (*r*(58) = 0.29, *p* < 0.05), and the MFPC during the go/no-go and left PSFC during the missing scan task (*r*(58) = 0.36, *p* < 0.05). Detailed results for the correlation statistics are shown in Supplementary Materials (Tables S1–S3). Therefore, Hypothesis 2 is not supported by this study.

Finally, the prefrontal activations across tasks were compared. Two-way ANOVA analyses on HbO data revealed a significant main effect of the task, *F* (2, 89) = 7.6, *p* = 0.001. Post hoc analyses using the Bonferroni method revealed that the participants showed strong activation during the missing scan task compared to the DCCS task (*p* < 0.01). Prefrontal activation did not differ between the go/no-go and other tasks (*p*s > 0.05). No significant effect of regions (*F* (8, 89) = 1.88, *p* = 0.08) nor a significant interaction between task and regions (*F* (16, 89) = 1.6, *p* = 0.08) were found. Therefore, Hypothesis 3 is supported by this study.

## 4. Discussion

This is the first study to examine the applicability of the unity–diversity model of EF in Chinese preschoolers, using both behavioral and neuroimaging approaches. It provides behavioral evidence to support the correlation between working memory, cognitive shifting, and inhibitory control, and the fNIRS evidence proves that the prefrontal activations for working memory tasks correlate with those for cognitive shifting and inhibitory control. However, both behavioral and neuroimaging results do not demonstrate a significant correlation between cognitive shifting and inhibitory control. This section discusses these findings and the limitations of this study.

### 4.1. Working Memory as the Common Executive Process of EF

The results showed that the working memory task was correlated with the cognitive shifting and inhibitory control tasks behaviorally. Still, performance on the latter two tasks did not correlate. This finding indicated that working memory might serve as a “foundation” for successful performance in cognitive shifting and inhibitory control tasks, which require the children to maintain and manipulate the rules in mind [64]. First, for the cognitive shifting task, even though memory demands were minimized by the experimenter reminding the participants of the current sorting criterion on each trial [65], they almost succeeded in the switching task (mean correct rate of 97%); it seems that cognitive shifting required working memory in addition to the ability to shift. This finding provides empirical evidence to support Garon’s hypothesis [19,66].

Second, for the correlation between the inhibition and working memory task, this finding provides empirical evidence to settle the arguments about whether inhibition is separate from working memory [67,68], whether inhibition is a behavioral product of exercising working memory [69], or whether working memory and inhibition depend on the same limited-capacity system so that increasing the demand on either affects one’s ability to do the other [70,71]. However, as they are significantly correlated, it is hard to cut the linkage between working memory and inhibitory control. Instead, improved working memory is associated with increased inhibition. This implies that working memory plays an important role in the whole EF process, and in other words, it might serve as the common executive process shared by all EF tasks. It also indicates that working memory is fundamental to concept learning [72] and is a critical factor in cognitive development [73,74]. The following section will elaborate more on this.

### 4.2. Applicability of the Unity–Diversity Model

First, the fNIRS results were similar to the behavioral results in this study: there was a medium correlation in the prefrontal activation between the working memory and cognitive shifting task and between the working memory and inhibitory control task. This finding corroborates the meta-analysis using activation likelihood estimation, which found the existence of partially separable but partially overlapping processes in children over 6 years [8]. The current study further extended this finding by demonstrating that there are both unity (working memory as the common executive process) and diversity (shifting does not correlate with inhibition) of EF in Chinese preschoolers (ages 4–6). Furthermore, there is growing evidence from neuroimaging studies suggesting a core network responsible for maintaining task sets, such as holding-in-mind, which seems to emerge early in life and is the prototype of working memory [75]. Therefore, the behavioral and neuroimaging evidence jointly proved the unity part of the unity–diversity model in preschoolers.

Second, this study also assessed whether the prefrontal activations differed across the shifting, inhibition, and updating tasks. The fNIRS results showed that preschoolers showed strong activation during the working memory task compared to the cognitive shifting task, indicating that the lateral prefrontal regions may be involved differently in the shifting and working memory tasks. Still, the relative recruitment of those brain regions may differ across different executive tasks [51]. Furthermore, such a difference might also stem from varying levels of cognitive challenge stemming from the two tasks, with almost all the participants performing successfully in the shifting task (mean correct rate 97%) but only a little more than half performing well in the updating task (mean correct rate 62%). Future studies will design different behavioral tasks to examine the neural correlates during these three executive processes. In addition, a non-significant relationship was found between cognitive shifting and inhibitory control, indicating that the two factors might be dissociable in the preschool years. This finding is generally consistent with a recent behavioral study, which found that the one-factor model was not statistically better (though adequate model fit) than the two-factor model consisting of two distinguishable factors [20]. Thus, although the present study did not use confirmatory factor analysis because of the limited number of EF tasks, the results are similar to the previous evidence. Therefore, these findings jointly proved the diversity part of the unity–diversity model of EF.

## 5. Conclusions

This study examined the relationship among the three domains (cognitive shifting, inhibitory control, and working memory) of EF to test the applicability of the unity–diversity model in preschoolers, using both behavioral and neural approaches. The behavioral results indicated that working memory correlated with cognitive shifting and inhibitory control, but the latter two did not correlate with each other, and the fNIRS results demonstrated that the prefrontal activations during the working memory task correlated with those during the cognitive shifting and inhibitory control. However, the latter two tasks still did not correlate with each other. These findings jointly indicated that there might be both unity (working memory as the common process) and diversity (shifting and inhibitory are separate) in preschoolers’ EF, supporting the unity–diversity model of EF proposed by Miyake and Friedman [6].

However, this study has some noticeable limitations. First, due to the limited time preschoolers could participate in this fNIRS study, there was only one task for each EF process, which might not comprehensively measure the target variables. Furthermore, the three tasks were performed in a fixed order, which might affect the participants’ performance compared with if they were randomized. Second, this study assessed only the lateral prefrontal regions with fNIRS. Other regions, such as the parietal regions, may also contribute to the executive process in a different manner. Future studies should include more regions if the fNIRS instrument has more channels (i.e., 32 × 32). Finally, the number of participants of different ages was small, and not enough to detect the age effect or pattern of executive function. Despite the limitations, this study contributes to the debate on the unity and diversity of the three constructs of executive function at both behavioral and neural levels in young children.

## Figures and Tables

**Table 1:** Mean (*SD*) of Accuracy in Each Task and their Correlations.

Task	M (%) (SD)	1	2	3
1. DCCS	0.97 (0.00)	-		
2. Go/No-Go	0.93 (0.01)	0.13	-	
3. Missing Scan	0.62 (0.05)	0.26 *	0.53 ***	-

Note. * *p* < 0.05, *** *p* < 0.001.

**Table 2:** Mean (*SD*) of Changes in HbO during The Task Phases after Subtracting Rest Phases.

ROI	DCCS	Go/No-Go	Missing Scan
left VLPFC	−0.02 (0.08)	−0.01 (0.04)	0.01 (0.06)
left VLPFC	0.01 (0.13)	0.03 (0.07)	−0.00 (0.01)
left DLPFC	0.00 (0.11)	−0.00 (015)	0.01 (0.03)
left DLPFC	−0.00 (0.12)	−0.06 (0.04)	0.03 (0.07)
left PSFC	−0.08 (0.18)	−0.03 (0.07)	0.06 (0.04)
left PSFC	−0.09 (0.13)	−0.07 (0.05)	−0.01 (0.05)
left TC	−0.03 (0.17)	−0.01 (0.01)	0.00 (0.04)
left TC	−0.05 (0.12)	−0.14 (0.05)	0.03 (0.03)
MFPC	−0.02 (0.13)	−0.01 (0.04)	0.02 (0.02)

Note. VLPFC = ventrolateral prefrontal cortex (VLPFC); DLPFC = dorsolateral prefrontal cortex (DLPFC); PSFC = posterior superior frontal cortex (PSFC); TC = temporal cortex (TC); MPFC = medial prefrontal cortex (MPFC).

## Data Availability

All the data for this study are available upon request.

## Supplementary Materials

The following supporting information can be downloaded at: https://www.mdpi.com/article/10.3390/brainsci12121722/s1, Table S1: Comparison of HbO Activations in Different Regions between DCCS and Go/No-Go Task; Table S2: Comparison of HbO Activations in Different Regions between DCCS and Missing Scan Task; Table S3: Comparison of HbO Activations in Different Regions between Go/No-Go and Missing Scan Task.
