# Supplementary material for: An fNIRS Study of Applicability of the Unity–Diversity Model of Executive Functions in Preschoolers
Source: Brain Sci. 2022 Dec 16;12(12):1722. doi: 10.3390/brainsci12121722 (PMC9776044; doi:10.3390/brainsci12121722)
Supplement: Supplementary file 1 [file brainsci-12-01722-s001.zip › brainsci-2027716-supplementary.pdf]

Supplementary Materials

Table S1. Comparison of HbO Activations in Different Regions between DCCS and Go/No-Go Task.

ROI (DCCS)	ROI (Go/No-Go)	r	*p*
left VLPFC	left VLPFC	0.01	0.96
left VLPFC	right VLPFC	−0.16	0.24
left VLPFC	left DLPFC	**−0.30**	**0.02**
left VLPFC	right DLPFC	0.04	0.78
left VLPFC	left PSFC	0.07	0.63
left VLPFC	right PSFC	0.21	0.14
left VLPFC	right TC	−0.22	0.11
left VLPFC	left TC	0.05	0.70
left VLPFC	MFPC	−0.06	0.67
right VLPFC	left VLPFC	−0.14	0.31
right VLPFC	right VLPFC	−0.13	0.35
right VLPFC	left DLPFC	−0.09	0.51
right VLPFC	right DLPFC	−0.15	0.28
right VLPFC	left PSFC	−0.15	0.33
right VLPFC	right PSFC	0.04	0.77
right VLPFC	right TC	−0.03	0.84
right VLPFC	left TC	−0.19	0.16
right VLPFC	MFPC	−0.08	0.53
left DLPFC	left VLPFC	−0.01	0.92
left DLPFC	right VLPFC	**−0.34**	**0.01**
left DLPFC	left DLPFC	−0.02	0.91
left DLPFC	right DLPFC	−0.16	0.24
left DLPFC	left PSFC	−0.18	0.22
left DLPFC	right PSFC	0.01	0.93
left DLPFC	right TC	−0.13	0.34
left DLPFC	left TC	**−0.28**	**0.04**
left DLPFC	MFPC	−0.19	0.15
right DLPFC	left VLPFC	0.10	0.49
right DLPFC	right VLPFC	−0.06	0.65
right DLPFC	left DLPFC	−0.18	0.19
right DLPFC	right DLPFC	−0.06	0.64
right DLPFC	left PSFC	0.07	0.66
right DLPFC	right PSFC	0.16	0.29
right DLPFC	right TC	−0.20	0.15
right DLPFC	left TC	−0.12	0.38
right DLPFC	MFPC	−0.03	0.81
left PSFC	left VLPFC	0.18	0.24
left PSFC	right VLPFC	0.11	0.49
left PSFC	left DLPFC	0.05	0.75
left PSFC	right DLPFC	0.02	0.90
left PSFC	left PSFC	0.23	0.13
left PSFC	right PSFC	0.12	0.43
left PSFC	right TC	−0.01	0.97
left PSFC	left TC	0.11	0.45
left PSFC	MFPC	0.29	0.05
right PSFC	left VLPFC	0.05	0.75

right PSFC	right VLPFC	0.08	0.59
right PSFC	left DLPFC	−0.26	0.06
right PSFC	right DLPFC	−0.10	0.52
right PSFC	left PSFC	−0.24	0.12
right PSFC	right PSFC	0.24	0.10
right PSFC	right TC	−0.09	0.52
right PSFC	left TC	−0.06	0.67
right PSFC	MFPC	0.03	0.84
right TC	left VLPFC	0.08	0.57
left TC	left VLPFC	0.11	0.43
left TC	right VLPFC	0.12	0.38
left TC	left DLPFC	0.24	0.07
left TC	right DLPFC	−0.09	0.49
left TC	left PSFC	0.07	0.67
left TC	right PSFC	0.23	0.11
left TC	right TC	0.09	0.52
left TC	left TC	0.19	0.16
left TC	MFPC	**0.30**	**0.02**
right TC	left VLPFC	0.08	0.57
right TC	right VLPFC	0.05	0.73
right TC	left DLPFC	−0.05	0.70
right TC	right DLPFC	0.02	0.87
right TC	left PSFC	−0.24	0.11
right TC	right PSFC	−0.13	0.37
right TC	right TC	−0.07	0.58
right TC	left TC	0.12	0.38
MFPC	left VLPFC	−0.01	0.92
MFPC	right VLPFC	−0.14	0.28
MFPC	left DLPFC	−0.20	0.14
MFPC	right DLPFC	−0.02	0.88
MFPC	left PSFC	−0.15	0.33
MFPC	right PSFC	0.06	0.67
MFPC	right TC	−0.25	0.06
MFPC	left TC	−0.17	0.20
MFPC	MFPC	−0.11	0.43

Note. Bold indicates significant results. VLPFC = ventrolateral prefrontal cortex (VLPFC); DLPFC = dorsolateral prefrontal cortex (DLPFC); PSFC = posterior superior frontal cortex (PSFC); TC = temporal cortex (TC); MPFC = medial prefrontal cortex (MPFC).

Table S2. Comparison of HbO Activations in Different Regions between DCCS and Missing Scan Task.

ROI (DCCS)	ROI (Missing Scan)	r	*p*
left VLPFC	left VLPFC	**0.28**	**0.03**
left VLPFC	right VLPFC	**0.37**	**0.01**
left VLPFC	left DLPFC	0.05	0.70
left VLPFC	right DLPFC	**0.32**	**0.02**
left VLPFC	left PSFC	0.18	0.23
left VLPFC	right PSFC	0.19	0.19
left VLPFC	right TC	−0.21	0.11
left VLPFC	left TC	0.12	0.39
left VLPFC	MFPC	0.13	0.33
right VLPFC	left VLPFC	−0.09	0.53
right VLPFC	right VLPFC	0.13	0.34
right VLPFC	left DLPFC	−0.11	0.41
right VLPFC	right DLPFC	0.24	0.07
right VLPFC	left PSFC	0.01	0.95
right VLPFC	right PSFC	0.06	0.70
right VLPFC	right TC	0.09	0.50
right VLPFC	left TC	0.20	0.13
right VLPFC	MFPC	0.09	0.49
left DLPFC	left VLPFC	0.07	0.59
left DLPFC	right VLPFC	0.20	0.14
left DLPFC	left DLPFC	0.12	0.38
left DLPFC	right DLPFC	0.21	0.14
left DLPFC	left PSFC	0.16	0.28
left DLPFC	right PSFC	0.12	0.40
left DLPFC	right TC	0.01	0.97
left DLPFC	left TC	0.14	0.30
left DLPFC	MFPC	−0.15	0.28
right DLPFC	left VLPFC	0.07	0.60
right DLPFC	right VLPFC	0.20	0.14
right DLPFC	left DLPFC	−0.23	0.09
right DLPFC	right DLPFC	**0.38**	**0.00**
right DLPFC	left PSFC	0.18	0.24
right DLPFC	right PSFC	0.25	0.09
right DLPFC	right TC	−0.02	0.86
right DLPFC	left TC	0.16	0.23
right DLPFC	MFPC	0.13	0.33
left PSFC	left VLPFC	−0.03	0.82
left PSFC	right VLPFC	−0.13	0.38
left PSFC	left DLPFC	0.21	0.16
left PSFC	right DLPFC	0.11	0.48
left PSFC	left PSFC	0.29	0.05
left PSFC	right PSFC	−0.17	0.27
left PSFC	right TC	−0.20	0.19
left PSFC	left TC	0.17	0.27
left PSFC	MFPC	−0.01	0.96
right PSFC	left VLPFC	−0.15	0.29
right PSFC	right VLPFC	−0.10	0.50
right PSFC	left DLPFC	−0.11	0.46
right PSFC	right DLPFC	**−0.29**	**0.05**

right PSFC	left PSFC	0.16	0.31
right PSFC	right PSFC	0.08	0.56
right PSFC	right TC	0.17	0.25
right PSFC	left TC	0.24	0.09
right PSFC	MFPC	0.11	0.45
right TC	left VLPFC	0.09	0.49
left TC	left VLPFC	0.08	0.58
left TC	right VLPFC	0.03	0.81
left TC	left DLPFC	0.00	0.97
left TC	right DLPFC	0.04	0.77
left TC	left PSFC	0.24	0.11
left TC	right PSFC	−0.12	0.42
left TC	right TC	−0.16	0.22
left TC	left TC	0.24	0.07
left TC	MFPC	0.25	0.06
right TC	left VLPFC	0.09	0.49
right TC	right VLPFC	0.03	0.81
right TC	left DLPFC	**0.29**	**0.03**
right TC	right DLPFC	−0.11	0.44
right TC	left PSFC	−0.02	0.92
right TC	right PSFC	0.02	0.91
right TC	right TC	0.12	0.36
right TC	left TC	0.13	0.32
MFPC	left VLPFC	−0.05	0.70
MFPC	right VLPFC	−0.01	0.95
MFPC	left DLPFC	−0.13	0.33
MFPC	right DLPFC	0.15	0.26
MFPC	left PSFC	0.10	0.53
MFPC	right PSFC	−0.01	0.96
MFPC	right TC	0.03	0.80
MFPC	left TC	0.09	0.51
MFPC	MFPC	0.03	0.82

Note. Bold indicates significant results. VLPFC = ventrolateral prefrontal cortex (VLPFC); DLPFC = dorsolateral prefrontal cortex (DLPFC); PSFC = posterior superior frontal cortex (PSFC); TC = temporal cortex (TC); MPFC = medial prefrontal cortex (MPFC).

Table S3. Comparison of HbO Activations in Different Regions between Go/No-Go and Missing Scan Task.

ROI (Go/No-Go)	ROI (Missing Scan)	r	*p*
left VLPFC	left VLPFC	0.08	0.56
left VLPFC	right VLPFC	0.09	0.52
left VLPFC	left DLPFC	0.05	0.73
left VLPFC	right DLPFC	0.14	0.32
left VLPFC	left PSFC	0.26	0.08
left VLPFC	right PSFC	−0.09	0.53
left VLPFC	right TC	0.04	0.75
left VLPFC	left TC	0.07	0.62
left VLPFC	MFPC	0.09	0.52
right VLPFC	left VLPFC	−0.15	0.28
right VLPFC	right VLPFC	0.14	0.29
right VLPFC	left DLPFC	−0.04	0.79
right VLPFC	right DLPFC	−0.17	0.21
right VLPFC	left PSFC	0.28	0.07
right VLPFC	right PSFC	−0.09	0.56
right VLPFC	right TC	0.11	0.41
right VLPFC	left TC	0.16	0.23
right VLPFC	MFPC	0.25	0.07
left DLPFC	left VLPFC	0.00	0.99
left DLPFC	right VLPFC	−0.24	0.08
left DLPFC	left DLPFC	**0.33**	**0.01**
left DLPFC	right DLPFC	−0.06	0.69
left DLPFC	left PSFC	0.06	0.68
left DLPFC	right PSFC	0.10	0.47
left DLPFC	right TC	−0.01	0.96
left DLPFC	left TC	0.10	0.45
left DLPFC	MFPC	−0.17	0.21
right DLPFC	left VLPFC	−0.18	0.18
right DLPFC	right VLPFC	0.07	0.60
right DLPFC	left DLPFC	−0.07	0.63
right DLPFC	right DLPFC	0.05	0.69
right DLPFC	left PSFC	−0.03	0.87
right DLPFC	right PSFC	−0.26	0.07
right DLPFC	right TC	−0.07	0.61
right DLPFC	left TC	−0.11	0.42
right DLPFC	MFPC	0.01	0.96
left PSFC	left VLPFC	0.03	0.87
left PSFC	right VLPFC	−0.10	0.50
left PSFC	left DLPFC	0.08	0.58
left PSFC	right DLPFC	0.07	0.65
left PSFC	left PSFC	−0.20	0.19
left PSFC	right PSFC	−0.05	0.77
left PSFC	right TC	**−0.48**	**0.00**
left PSFC	left TC	−0.20	0.19
left PSFC	MFPC	−0.15	0.31
right PSFC	left VLPFC	0.00	0.97
right PSFC	right VLPFC	0.04	0.78
right PSFC	left DLPFC	−0.06	0.66

right PSFC	right DLPFC	−0.07	0.64
right PSFC	left PSFC	**0.38**	**0.01**
right PSFC	right PSFC	0.02	0.89
right PSFC	right TC	0.05	0.72
right PSFC	left TC	**0.29**	**0.04**
right PSFC	MFPC	0.00	1.00
left TC	left VLPFC	−0.00	0.99
left TC	right VLPFC	−0.01	0.96
left TC	left DLPFC	−0.01	0.93
left TC	right DLPFC	−0.06	0.64
left TC	left PSFC	0.15	0.30
left TC	right PSFC	−0.05	0.71
left TC	right TC	−0.23	0.08
left TC	left TC	0.13	0.31
left TC	MFPC	0.22	0.10
right TC	left VLPFC	−0.17	0.20
right TC	right VLPFC	0.09	0.52
right TC	left DLPFC	−0.10	0.45
right TC	right DLPFC	0.01	0.93
right TC	left PSFC	−0.16	0.29
right TC	right PSFC	−0.20	0.15
right TC	right TC	0.07	0.60
right TC	left TC	−0.15	0.25
right TC	MFPC	−0.07	0.62
MFPC	left VLPFC	−0.16	0.23
MFPC	right VLPFC	0.00	1.00
MFPC	left DLPFC	−0.04	0.76
MFPC	right DLPFC	0.01	0.94
MFPC	left PSFC	**0.36**	**0.01**
MFPC	right PSFC	−0.20	0.17
MFPC	right TC	0.11	0.40
MFPC	left TC	0.16	0.24
MFPC	MFPC	0.23	0.09

Note. Bold indicates significant results. VLPFC = ventrolateral prefrontal cortex (VLPFC); DLPFC = dorsolateral prefrontal cortex (DLPFC); PSFC = posterior superior frontal cortex (PSFC); TC = temporal cortex (TC); MPFC = medial prefrontal cortex (MPFC).
